# Evaluating Fc-mediated antibody functions against SARS-CoV-2 in PLHIV: reduced FcγRIIIa-dependent activity and ADCC despite preserved neutralising antibodies

**DOI:** 10.3389/fimmu.2026.1932685

**Published:** 2026-09-24

**Authors:** Swarali Kurle, Pratiksha Pansambal, Madhuri Thakar, Manisha Ghate, Shubhangi Bichare, Pallavi Shidhaye, Sumit Aggarwal

**Affiliations:** 1 HIV Drug Resistance Laboratory - ICMR - National Institute of Translational Virology and AIDS Research, Pune, Maharashtra, India; 2 Division of Immunology and Serology - ICMR - National Institute of Translational Virology and AIDS Research, Pune, Maharashtra, India; 3 Division of Clinical Sciences - ICMR - National Institute of Translational Virology and AIDS Research, Pune Maharashtra, India; 4 Division of Descriptive Research- Indian Council of Medical Research, , New Delhi, India

**Keywords:** ADCC, ART, PLHIV, SARS-CoV-2, Spike

## Abstract

**Background:**

Antibody-dependent cellular cytotoxicity (ADCC) is a critical Fc-mediated immune mechanism contributing to antiviral defence. However, its functionality in people living with HIV (PLHIV) following SARS-CoV-2 exposure remains poorly understood.

**Objective:**

This study evaluates the anti-Spike ADCC antibody levels, functional ADCC activity, and neutralising antibody responses in HIV-negative and HIV-positive individuals.

**Methodology:**

The study included 30 samples per group. Anti-Spike ADCC-binding antibodies were measured using FcγRIIIa-binding assays, while functional ADCC activity was evaluated by rapid fluorometric antibody-dependent cellular cytotoxicity (RFADCC) assays. Neutralising antibody responses were assessed using surrogate virus neutralization assays.

**Results:**

HIV-negative Spike-positive individuals exhibited significantly higher levels of ADCC antibodies compared to HIV-positive counterparts (fold response: 8.74 vs 6.23; p=0.0056). Consistently, functional ADCC activity was reduced in HIV-positive individuals (mean: 43.87%) compared to HIV-negative individuals (mean: 52.16%; p=0.03), indicating impaired Fc-mediated effector function. In contrast, neutralising antibody responses were largely preserved, with no significant difference observed between HIV-positive and HIV-negative Spike-positive groups, despite increased variability in the HIV-positive cohort.

**Conclusion:**

These findings demonstrate reduced FcγRIIIa-dependent antibody activity and functional ADCC responses in PLHIV despite relatively preserved neutralising antibody responses, highlighting differences between Fc-mediated and neutralising antibody functions.

## 1 Introduction

Since December 2019, the severe acute respiratory syndrome coronavirus 2 (SARS-CoV-2) has emerged and spread rapidly throughout the world, raising serious concerns about its effects on people living with HIV (PLHIV). The Centers for Disease Control and Prevention (CDC) noted in March 2020 that PLHIV might be more vulnerable to serious COVID-19-related problems than the general population because of their impaired immune systems. When assessing the intersection of the HIV and COVID-19 pandemics, it is important to take into account that the PLHIV represent a very heterogeneous group with varied degrees of immunological competence (1, 2). This immunological diversity influences susceptibility to SARS-CoV-2 infection, disease severity, and response to therapeutic interventions and vaccines (3). The extent of immune dysfunction in PLHIV is closely linked to antiretroviral therapy (ART) status, as individuals not receiving ART are at a higher risk of opportunistic infections due to impaired immune function (4). Emerging evidence suggests that uncontrolled HIV infection, particularly in individuals with CD4 counts below 200 cells/mm³, is associated with adverse COVID-19 outcomes (5, 6). These observations underscore the need to understand both humoral and cellular immune responses to SARS-CoV-2 in PLHIV.

Current knowledge of antibody-mediated immunity against SARS-CoV-2 is largely focused on neutralising antibodies (NAbs) directed against the viral spike (S) protein, which play a critical role in protection following natural infection or vaccination (7, 8). NAbs are typically detectable within two weeks of disease onset in recovered individuals. However, the emergence of multiple SARS-CoV-2 variants has raised concerns about the durability and breadth of neutralising responses, as antibody responses can persist for months after infection, their magnitude and durability vary substantially between individuals (9–11). Beyond neutralization, spike-specific antibodies can mediate antiviral effects through Fc-dependent effector functions, including antibody-dependent cellular cytotoxicity (ADCC), antibody-dependent cellular phagocytosis (ADCP), and complement-dependent cytotoxicity (12–15). Among these, ADCC plays a crucial role in viral clearance by facilitating the elimination of infected cells (16, 17). In this process, antibodies bind to viral antigens expressed on the surface of infected cells and engage Fc receptors on effector cells, particularly natural killer (NK) cells, leading to targeted cytotoxicity. ADCC responses have been shown to contribute significantly to immune control in several viral infections, including hepatitis B virus, HIV, and influenza (18–20). Recent studies have demonstrated robust ADCC responses against SARS-CoV-2 in infected individuals, highlighting their importance in antiviral immunity (17, 21, 22). However, despite persistent immune dysregulation in PLHIV, Fc-mediated antibody functions against SARS-CoV-2 remain insufficiently explored, particularly in the Indian population. To the best of our knowledge, this is the first study to investigate SARS-CoV-2-specific ADCC responses in PLHIV from India. Addressing this critical knowledge gap, the present study evaluated SARS-CoV-2-specific ADCC responses in PLHIV. Our findings demonstrate that PLHIV exhibit significantly lower ADCC activity compared to HIV-uninfected individuals, suggesting impaired Fc-mediated antiviral immunity in this population.

## 2 Materials and methods

### 2.1 Study groups

The study was conducted in three groups, viz, HIV-infected individuals having antibodies against the spike protein.(HIV+SPIKE+, N = 30), individuals showing the presence of anti-spike antibodies but negative for HIV antibodies (HIV-SPIKE+, N = 30) and individuals negative for both HIV and anti-Spike antibodies (HIV-SPIKE-, N = 30). The HIV-Spike- group consisted of archived plasma samples collected during 2009–2010 for control panels negative for HIV, HBV and HCV. These plasma samples were collected from adult blood donors prior to the COVID-19 pandemic without any exposure to SARS-CoV-2. Hence, individual demographic information was not available for these archived samples. Demographic details of the study groups are given in **Table 1**. All PLHIVs were on ART with CD4 counts ranging from 42 to 1164 cells/mm³. The samples were collected and stored earlier from a study titled ‘Evaluation of Antibody-Dependent Cytotoxicity (ADCC) and Antibody-Dependent Enhancement (ADE) responses against SARS-CoV-2 infection’. The present study was approved by the institutional Ethics Committee (No. NARI-EC/2022-07).

**Table 1:** Demographic details of samples.

	HIV+SPIKE+	HIV-SPIKE+
Age (years)		
Median (Range)	46 (31-61)	39 (29-56)
Gender		
Male	13 (43.3%)	13 (43.3%)
Female	17 (56.6%)	17 (56.6%)
Sample distribution based on CD4 count		
<200 cells/mm^3^	4 (13.3%)	NA
200–499 cells/mm^3^	5 (16.6%)	NA
≥500 cells/mm^3^	21 (70%)	NA
ART Regimen:		
Tenofovir-Lamivudine-Dolutegravir (TLD)	30(100%)	NA

### 2.2 Detection of Anti-Spike antibodies

Plasma samples were thawed and assessed for the presence of anti-spike antibodies of SARS-CoV-2 using indirect ELISA by the ACRO Biosystems Spike kit (Cat #TAS-K025) following the kit instructions. Plasma samples, along with the kit-provided positive and negative controls, were diluted 1:400 in the supplied dilution buffer, and 100 µL of each diluted sample/control was added to the pre-coated Spike protein wells. The plates were incubated for 1 h at 37 °C, followed by three washes with 1× washing buffer. HRP-conjugated anti-human IgG was diluted 1:99 in the supplied dilution buffer, and 100 µL of the diluted conjugate was added to each well, followed by incubation for 1 h at 37 °C. After washing, 100 µL of substrate solution was added and incubated for 20 min at 37 °C in the dark. The reaction was stopped by adding 50 µL of stop solution, and absorbance was measured at 450 nm. The manufacturer’s recommended cut-off of OD450 = 0.1 was used to determine the presence of anti-Spike antibodies. Samples positive for anti-Spike antibodies were subsequently included for ADCC and neutralising antibody analyses.

### 2.3 Spike protein and cell line

The HEK-293T ACE 2 expressing cell line (NR-5251, kindly provided by BEI Resources Repository) was used to assess NK cell‐mediated target cell killing. Cells were cultured in complete medium (Dulbecco’s Modified Eagle Medium (DMEM) (Gibco-11995-065)) supplemented with 10% (v/v) foetal bovine serum (Gibco 26140-079). SARS CoV-2 spike protein (BEI Resources Repository NR-53524) was used for the ADCC assays.

### 2.4 Assessment of ADCC response

ADCC response was measured by two methods having different modes of ADCC detection: a) ADCC reporter bioassay, which detects the presence and relative level of FcγRIIIa-engaging, ADCC-mediating antibodies bound to the engineered effector cells and target cells expressing Spike protein; b) Rapid and Fluorometric Antibody-Dependent Cell-mediated Cytotoxicity (RFADCC), which measures the lysis of target cells.

#### 2.4.1 ADCC reporter bioassay

Anti-SARS-CoV-2 positive plasma samples were tested for the presence of anti-Spike antibodies capable of mediating FcγRIIIa-dependent ADCC using a commercial ADCC reporter bioassay (Promega, Cat. No. G7010) according to the manufacturer’s instructions. Briefly, S-CHO-K1 cells expressing SARS-CoV-2 Spike protein (Promega, Cat. No. CS3195A05) were seeded at 40,000 cells/well in 96-well white flat-bottom plates and incubated overnight at 37 °C with 5% CO_2_. The target cells were then incubated with plasma samples at a 1:10 dilution or without plasma for 30 min at 37 °C. Subsequently, 25 µL of the ADCC effector-cell suspension provided as part of the kit, consisting of engineered Jurkat cells expressing human FcγRIIIa V158, was added to each well according to the manufacturer’s protocol. The manufacturer’s protocol specifies the volume of the supplied effector-cell suspension but does not provide the absolute number or concentration of effector cells in the suspension. Therefore, a conventional effector-to-target (E:T) ratio could not be calculated. The assay was performed using the manufacturer-specified effector-cell volume and target-cell number. The plates were then incubated for 6 h at 37 °C. Following incubation, Bio-Glo™ luciferase reagent was added to each well and the plates were incubated for 10–15 min at room temperature. Luminescence was measured using a Multimode Plate Reader (VICTOR X2, PerkinElmer).


$$\text{Fold response}=\frac{\text{RLU of antibody containing sample}}{\text{RLU of no antibody control}}$$

The engineered Jurkat effector cells supplied with the kit express human FcγRIIIa and are activated following engagement of the Fc portion of antibodies bound to Spike-expressing target cells, resulting in NFAT-mediated luciferase expression. Therefore, this assay was used to assess the presence and relative level of FcγRIIIa-engaging, ADCC-mediating antibodies rather than directly measuring target-cell lysis (**Figure 1A**).

**Figure 1:**
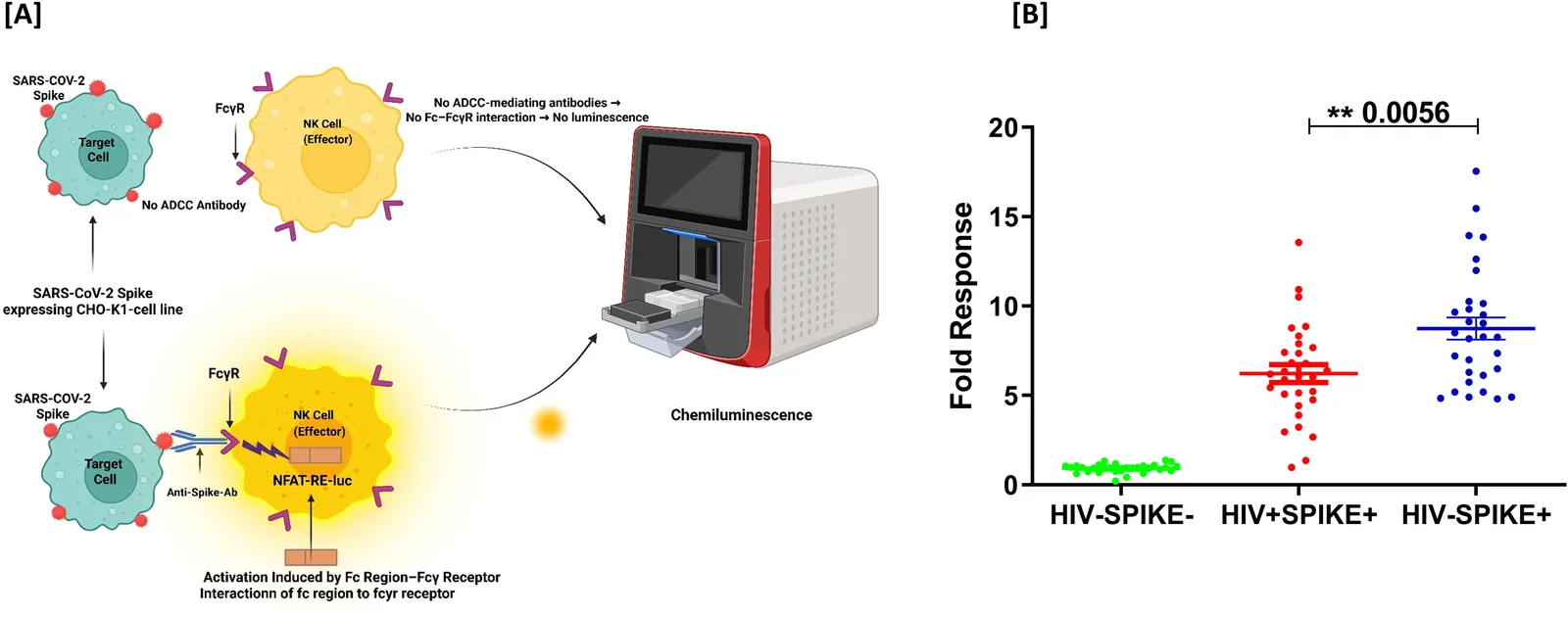
ADCC response by reporter bioassay. **(A)**Schematic of the ADCC assay where anti-spike antibodies bind SARS-CoV-2 spike–expressing target cells and engage FcγR-expressing reporter effector cells, triggering NFAT-driven luciferase expression measured as chemiluminescence. **(B)**The scatter plots illustrate the presence of ADCC mediating antibodies (expressed in terms of fold response) in HIV-SPIKE- (n = 30), HIV-SPIKE+ (n = 30), and HIV+SPIKE+ individuals (n = 30) (X-axis). The line represents the mean ± SD. Statistical significance was assessed using the unpaired Mann–Whitney U test, with P < 0.05 considered statistically significant.

#### 2.4.2 Rapid and fluorometric antibody-dependent cell-mediated cytotoxicity assay

ADCC activity against Spike-expressing target cells was assessed using the rapid and fluorometric antibody-dependent cell-mediated cytotoxicity (RFADCC) assay as described previously (23, 24) (**Figure 2A**). HEK293T-ACE2 cells were used as target cells. The target cells (1 × 10^6^ cells/mL) were pulsed with recombinant SARS-CoV-2 Spike protein (5 µg/10^6^ cells) for 1 h at room temperature. HEK293T-ACE2 cells without Spike protein were used as a negative target-cell control. Following incubation, cells were washed with ice-cold PBS and subsequently resuspended in 300 µL of Diluent C buffer (Sigma, Cat. No. CGLDIL). These target cells were then labelled with PKH-26 dye (Sigma, Cat. No. P9691) according to the manufacturer’s instructions and washed once with ice-cold PBS to remove excess dye. Subsequently labelled with CFSE [5-(and-6)-carboxyfluorescein diacetate succinimidyl ester] for 3 min, followed by washing with ice cold PBS. Labelled target cells were finally resuspended in DMEM medium and dispensed into 96-well U-bottom plates at 2 × 10^4^ cells/well. Plasma samples (source of antibodies) were diluted 1:10 and added to the respective target-cell wells. Monoclonal antibody D-002 () was included as a positive control. Following this, the plates were incubated for 30 min. Peripheral Blood Mononuclear Cells (PBMCs) obtained from a healthy HIV-uninfected donor were used as effector cells. The effector cells were added at a 10:1 E:T ratio at 2 × 10^5^ cells/well. The plates were then incubated for 4 h at 37 °C. The cells were subsequently stained with anti-CD3 antibody (BD Biosciences, Cat. No. 555335) and anti-CD14 antibody (BD Biosciences, Cat. No. 563561) and analysed on flowcytometer (BD FACSAria™ Fusion) within 24 h of staining (100,000 gated lymphocyte events per tube). The gating strategy is shown in **Figure 2B**. The data was analysed using FlowJo (version 10.8.1;FlowJo, LLC). The uptake of PKH-26-labelled target-cell fragments by CD14-positive monocytes was used as the RFADCC readout. The percentage of CD3-negative CD14-positive PKH-26-positive monocytes was interpreted as a surrogate measure of functional antibody-mediated effector activity.

**Figure 2:**
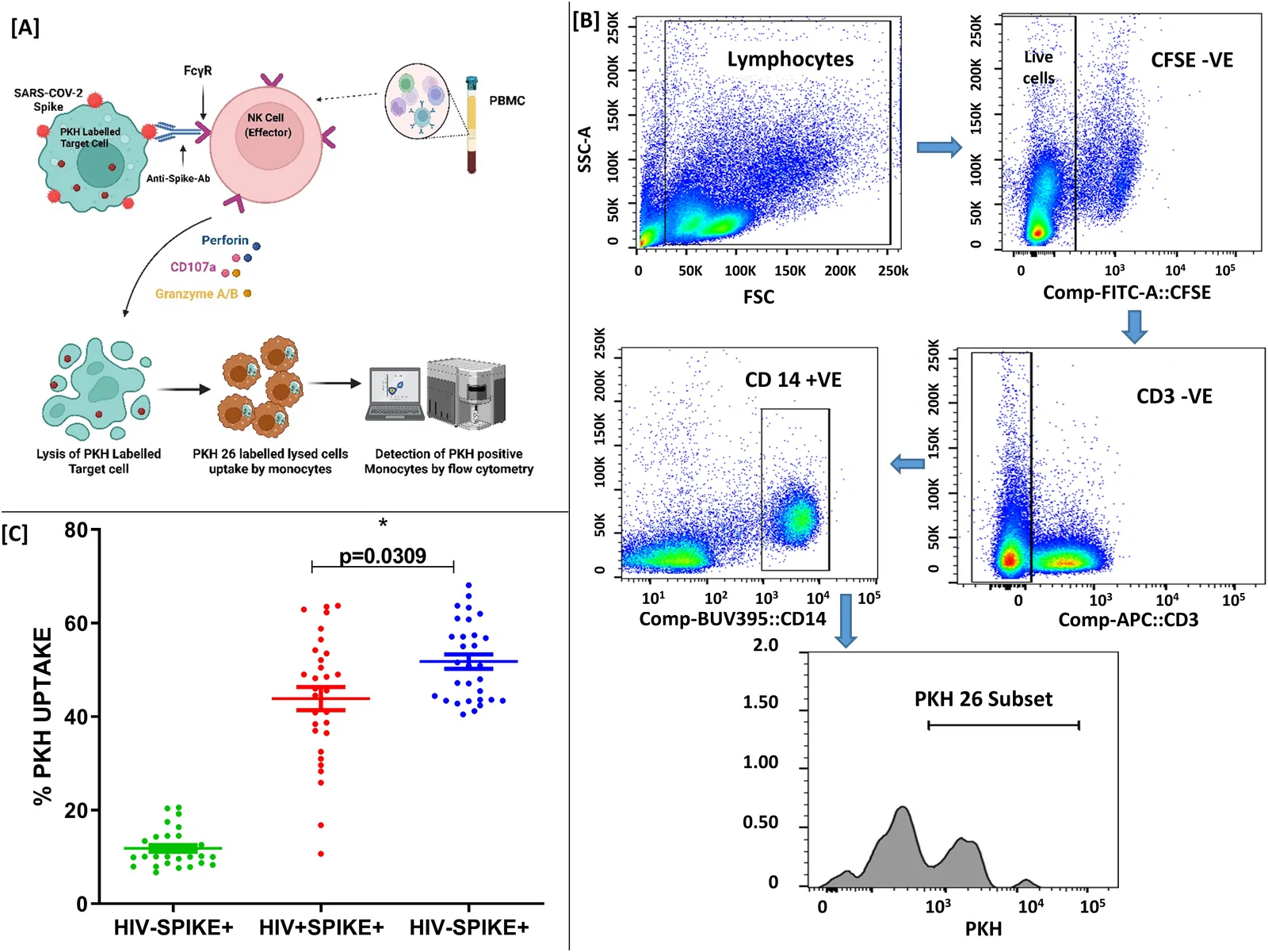
**(A)** Schematic representation of the RFADCC assay showing antibody-mediated target-cell lysis by FcγR-expressing effector cells, followed by uptake of PKH-26-labelled target-cell material by CD14+ monocytes. **(B)** Representative gating strategy used to identify CFSE-negative CD3-CD14+ monocytes, followed by identification/quantification of PKH26-positive cells. **(C)** Dot plots illustrating PKH-26-positive monocytes across the three study groups. The line represents the mean ± SD. Statistical comparisons were performed using the unpaired Mann–Whitney U test, with P < 0.05 considered statistically significant.

### 2.5 Detection of neutralising antibodies

The plasma samples were also tested for anti-SARS-CoV-2 spike-specific neutralising antibodies using the qualitative cPass SARS-CoV-2 Neutralisation Antibody Detection Kit (GenScript, Cat.no.L00847A) using the kit instructions. Briefly, 1:10 diluted plasma samples were incubated with 1:1000 diluted receptor-binding domain of spike protein(RBD)-HRP solution for 30 minutes at 37 °C. Then 100µL of the RBD-HRP Neutralisation Reaction Mixtures was transferred to a 96-well microplate coated with human ACE2 receptor. After 15 minutes of incubation at 37 °C, the plates were washed four times with wash buffer, 100 µl of TMB solution was added to each well, followed by incubation for 15 min at room temperature in the dark, and then 50 μl/well of stop solution was added to terminate the reaction (**Figure 3A**). Absorbance was measured at 450 nm using a VICTOR X2 Multimode Plate Reader (PerkinElmer). The data were analysed as follows,

**Figure 3:**
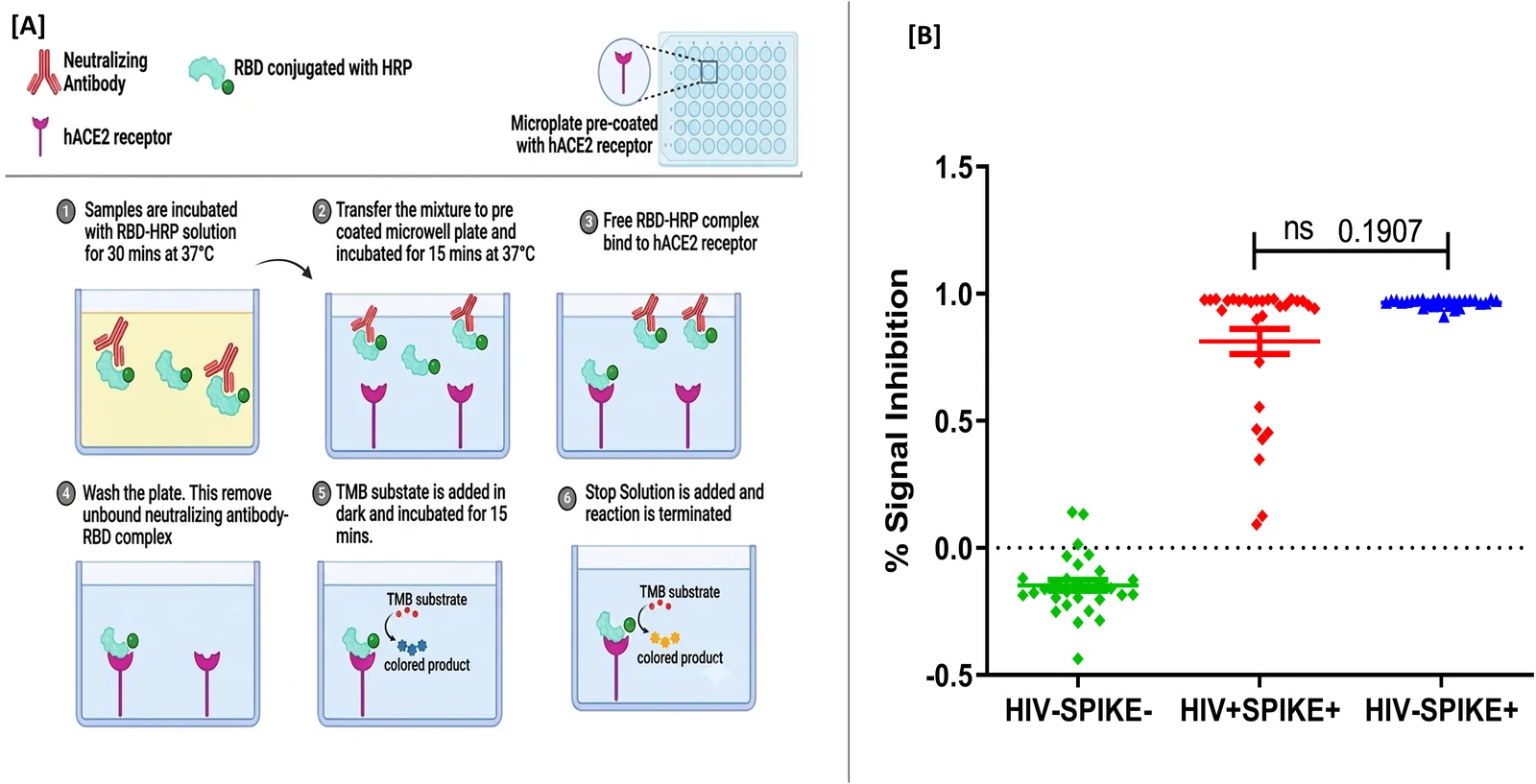
Surrogate neutralization assay. **(A)** Schematic representation of the cPass SARS-CoV-2 surrogate virus neutralization assay showing inhibition of the RBD–ACE2 interaction by neutralising antibodies present in plasma samples. **(B)** Scatter plot showing percentage signal inhibition in HIV-Spike-, HIV-Spike+, and HIV+Spike+ groups. The line represents the mean ± SD. Statistical significance was assessed using the unpaired Mann–Whitney test, with P < 0.05 considered statistically significant.


$$\%\text{Signal Inhibition}=[1–(\frac{\text{OD value of Sample}}{\text{OD value of Negative Control}})]\times 100$$

Cut off was % signal inhibition above 30 was conferred as the presence of neutralising antibodies against SARS-CoV-2.

### 2.6 Statistical analysis

Statistical analysis was performed with GraphPad Prism version 5.0 (Graph Pad Software, San Diego, CA). The nonparametric Mann–Whitney test was used to compare the presence and functionality of ADCC antibodies across the groups. A p-value of < 0.05 was considered to indicate a significant difference.

## 3 Results

### 3.1 HIV positive individuals showed lower levels of FcγRIIIa receptor binding ADCC antibodies with lower functionalities

Anti-Spike ADCC antibody levels were quantified across the HIV-SPIKE-(control), HIV-SPIKE+, and HIV+SPIKE+ groups (n = 30 per group). The HIV-SPIKE-group exhibited minimal background FcγRIIIa receptor-binding activity, with a fold response of 0.92 (range: 0.21–1.38), a 75th percentile of 1.049, and a lower 95% confidence interval (CI) of 0.824, confirming low background activity and assay specificity. In contrast, the HIV-SPIKE+ group demonstrated significantly higher levels of anti-Spike ADCC antibodies, with a fold response of 8.74 (range: 4.81–17.54), compared with 6.23 (range: 0.98–13.57) in the HIV+SPIKE+ group (p = 0.0056) (**Figure 1B**). The HIV-SPIKE+ group showed a 75th percentile of 7.606 and a lower 95% CI of 5.205 (SD = 2.748), whereas the HIV+SPIKE+ group showed a 75th percentile of 10.059 and a lower 95% CI of 7.485 (SD = 3.367). Thus, although anti-Spike ADCC antibodies were detectable in HIV-infected individuals, their FcγRIIIa receptor-binding levels were significantly lower than those observed in HIV-negative, Spike-positive individuals.

ADCC antibodies were further assessed for their functional capacity to mediate lysis of Spike-expressing target cells using the RFADCC assay. Following antibody-dependent target-cell lysis, PKH-26-labelled target-cell material is engulfed by CD14+ monocytes and detected as PKH-26-positive monocytes (**Figure 2C**). The HIV-SPIKE+ group demonstrated higher ADCC activity, with a mean of 52.16% (range: 41.2–68.1%), a 75th percentile of 57.4%, and a lower 95% CI of 49.01% (SD = 8.28). In comparison, the HIV+SPIKE+ group showed a mean ADCC activity of 43.87% (range: 10.7–63.7%), a 75th percentile of 53.15%, and a lower 95% CI of 38.80% (SD = 13.58). The difference between the HIV-SPIKE+ and HIV+SPIKE+ groups was statistically significant (p = 0.03), indicating reduced functional ADCC activity in HIV-infected individuals. The HIV-SPIKE-control group showed low background activity, with a mean frequency of 11.85% PKH-26-positive cells (range: 6.71–20.6%), a 75th percentile of 14.08%, and a lower 95% CI of 10.41%.

### 3.2 Anti-spike neutralising antibodies

Neutralising antibody responses were evaluated across the HIV-SPIKE-(control), HIV-SPIKE+, and HIV+SPIKE+ groups (n = 30 per group). The HIV-SPIKE-group showed minimal or no detectable neutralising activity, with a mean inhibition of -14.75% (range: -43.56 to 14.12%), a 75th percentile of -11.93%, and a lower 95% CI of -19.09%, consistent with the absence of SARS-CoV-2-specific neutralising antibodies in the control group. The HIV-SPIKE+ group demonstrated robust neutralising activity, with a mean inhibition of 96.45% (range: 91.14–97.99%), a 75th percentile of 97.39%, and a lower 95% CI of 95.88% (SD = 1.51%). In comparison, the HIV+SPIKE+ group showed a mean inhibition of 87.20% (range: 9.24–97.94%), with a 75th percentile of 97.60% and a lower 95% CI of 78.17% (SD = 24.16%) (**Figure 3B**). Although the HIV+SPIKE+ group showed greater variability in neutralising responses, the majority of individuals demonstrated high levels of inhibition. No statistically significant difference was observed between the HIV-SPIKE+ and HIV+SPIKE+ groups, indicating that overall SARS-CoV-2 neutralising antibody activity was preserved in HIV-infected individuals despite greater inter-individual variability.

## 4 Discussion

PLHIV may exhibit persistent immune dysregulation despite effective ART, which can influence the quality and functionality of immune responses to viral infections and vaccination (25). Several studies have shown that PLHIV receiving ART are able to develop SARS-CoV-2-specific binding and neutralising antibodies following infection or vaccination (22, 26–28). In agreement with these findings, we detected anti-Spike antibodies and neutralising antibodies in PLHIV in our study. Notably, neutralising antibody responses did not differ significantly between HIV-positive and HIV-negative Spike-positive individuals, indicating that the overall neutralising antibody response was largely preserved in our cohort. Neutralization, however, represents only one aspect of antibody-mediated antiviral immunity. In addition spike-specific antibodies can recruit immune effector mechanisms through their Fc regions, including ADCC, following interaction with Fc receptors on effector cells (12, 29, 30). In contrast to the relatively preserved neutralising response, we observed lower Fc-mediated antibody activity in PLHIV. The ADCC reporter assay showed significantly lower FcγRIIIa-dependent antibody activity among HIV-positive Spike-positive individuals than among their HIV-negative counterparts. A similar pattern was observed with the RFADCC assay, which also demonstrated lower functional ADCC activity in the HIV-positive group. These findings suggest that the differences between the two groups were more evident in Fc-mediated antibody function than in neutralising activity.

The concordant findings obtained using the two ADCC approaches are important because the assays provide complementary information. The reporter bioassay uses engineered Jurkat cells expressing FcγRIIIa and measures activation following engagement of FcγRIIIa by antibody Fc regions bound to Spike-expressing target cells. It therefore provides a standardized assessment of the ability of antibodies to engage FcγRIIIa. In contrast, the RFADCC assay uses primary PBMCs as effector cells and provides a functional cellular readout of antibody-mediated target-cell damage (23, 24, 26). The observation of reduced activity in both assays supports the presence of altered Spike-specific Fc-mediated antibody activity in PLHIV. However, these assays capture different aspects of ADCC, with the reporter assay reflecting FcγRIIIa-dependent antibody activity rather than directly measuring target-cell killing. The reduced Fc-mediated activity observed in PLHIV may potentially reflect differences in antibody characteristics or Fc receptor engagement. Previous studies have shown that antibody Fc glycosylation, subclass distribution, and Fc receptor interactions can influence Fc-mediated antiviral functions (29, 31). HIV infection has also been associated with alterations in B-cell maturation and antibody responses, including changes in germinal-centre responses during SARS-CoV-2 infection (32). These mechanisms could potentially contribute to the reduced Fc-mediated activity observed in our study. However, our study did not directly assess antibody Fc glycosylation, subclass distribution, Fc affinity, or other molecular characteristics. Thus, the observed reduction in ADCC activity cannot be conclusively attributed to altered qualitative properties of the antibodies. Rather, altered antibody characteristics and FcγRIIIa engagement represent possible mechanisms that require further investigation (29, 31).

The source of effector cells used in the RFADCC assay is an important consideration in the interpretation of these findings. This approach provided a standardized effector-cell environment for comparing the activity of antibodies from the different study groups. At the same time, this approach does not reflect the complete immune environment of PLHIV. HIV infection can alter the phenotype and function of NK cells and other Fc receptor-bearing cells, which may themselves influence Fc-mediated antiviral responses (30). Since effector cells from HIV-positive and HIV-negative individuals were not compared in this study, we cannot determine whether HIV-associated changes in NK-cell or other effector-cell function contributed to the observed differences. The results should therefore be interpreted primarily as differences in antibody-mediated Fc function under standardized assay conditions rather than as a direct measure of the overall ADCC capacity of PLHIV *in vivo*.

An important finding was the preservation of neutralising antibody responses despite the reduction in Fc-mediated activity. Neutralising and Fc-dependent antibody functions are complementary but distinct components of humoral immunity, and preservation of one does not necessarily imply preservation of the other. Previous studies have also shown that antibody levels and neutralising activity alone may not fully capture the functional characteristics of the antibody response in PLHIV (25, 33–35). Our findings therefore highlight the value of examining Fc-mediated functions alongside neutralising activity when assessing the quality of SARS-CoV-2-specific humoral responses in PLHIV.

We observed reduced ADCC activity among some participants with advanced CD4 depletion. Although this observation raises the possibility that greater immune depletion may be associated with impaired Fc-mediated responses, the number of individuals with CD4 counts below 200 cells/mm³ in our cohort was limited. Therefore, no definitive association between CD4 count and ADCC activity can be established from the present study. Larger studies with appropriate stratification according to CD4 count, viral suppression, and duration of ART will be required to investigate this relationship.

These findings should be interpreted in light of few limitations. The relatively small sample size and cross-sectional design limited detailed subgroup analyses and assessment of changes in antibody function over time. We did not assess Fc glycosylation, antibody subclass distribution, Fc receptor binding or affinity, or NK-cell phenotype and function, which could help explain the reduced Fc-mediated activity. In addition, variant-specific ADCC responses were not evaluated, which may be relevant given the ongoing antigenic evolution of SARS-CoV-2.

Importantly, the reduced ADCC observed in our study should not be interpreted as direct evidence of increased susceptibility to SARS-CoV-2 reinfection or reduced vaccine efficacy. Although Fc-dependent antibody functions may contribute to antiviral protection independently of neutralization, our study did not assess SARS-CoV-2 reinfection, breakthrough infection, vaccine effectiveness, disease severity, or other clinical outcomes. Therefore, these findings may have implications for understanding the quality of humoral immunity in PLHIV, but their relationship with clinical protection requires further investigation.

Overall, our findings demonstrate that PLHIV on ART with detectable SARS-CoV-2-specific antibodies can maintain relatively preserved neutralising antibody responses while exhibiting reduced FcγRIIIa-dependent antibody activity and functional ADCC responses. The findings suggest that Fc-mediated antibody functions may differ from neutralising activity in PLHIV and highlight the importance of evaluating multiple dimensions of antibody functionality. Further studies incorporating detailed Fc antibody profiling, assessment of autologous effector cells, longitudinal follow-up, and variant-specific antigens will be important to determine the mechanisms underlying altered Fc-mediated responses and their relevance to protective immunity against SARS-CoV-2 in PLHIV.

## 5 Conclusions

People living with HIV are particularly vulnerable to severe COVID-19 outcomes due to immune dysregulation and associated co-morbidities. The dual burden of HIV and SARS-CoV-2 infections poses significant challenges to treatment and management, necessitating targeted research to develop effective interventions. Our findings demonstrate that PLHIV with anti-SARS-CoV-2 spike antibodies have lower functional antibody-dependent cellular cytotoxicity responses as compared to the HIV negative population. The lower functionality of these responses in the HIV-infected group as compared to the uninfected group indicates the importance of assessment of restoration of immune response while understanding the effect of reinfections, new infections, and vaccination in this population. These insights advance our understanding of immune dynamics in PLHIV, underscore the importance of continued monitoring, and tailored therapeutic strategies to mitigate risks in this population.

## Data Availability

The raw data supporting the conclusions of this article will be made available by the authors, without undue reservation.

## References

[1] Augello M . Immunologic interplay between HIV/AIDS and COVID-19: Adding fuel to the flames? Curr HIV/AIDS Rep. (2023) 20:51–75. doi: 10.1007/s11904-023-00647-z 36680700 PMC9860243

[2] Hoft MA Burgers WA Riou C . The immune response to SARS-CoV-2 in people with HIV. Cell Mol Immunol. (2024) 21:184–96. 10.1038/s41423-023-01087-w PMC10806256 37821620

[3] Yang Y Iwasaki A . Impact of chronic HIV infection on SARS-CoV-2 infection, COVID-19 disease and vaccines. Curr HIV/AIDS Rep. (2022) 19:5–16. doi: 10.1007/s11904-021-00590-x 34843064 PMC8628277

[4] Mellors JW . Plasma viral load and CD4+ lymphocytes as prognostic markers of HIV-1 infection. Ann Intern Med. (1997) 126:946–54. doi: 10.7326/0003-4819-126-12-199706150-00003 9182471

[5] Dandachi D . Characteristics, comorbidities, and outcomes in a multicenter registry of patients with human immunodeficiency virus and coronavirus disease 2019. Clin Infect Dis. (2021) 73:e1964–72. doi: 10.1093/cid/ciaa1339 32905581 PMC7499544

[6] Calza L . COVID-19 outcomes in patients with uncontrolled HIV-1 infection. J Acquir Immune Defic Syndr. (2021) 86:e15–7. doi: 10.1097/qai.0000000000002537 33065583 PMC7727317

[7] Fielding CA . SARS-CoV-2 host-shutoff impacts innate NK cell functions, but antibody-dependent NK activity is strongly activated through non-spike antibodies. Elife. (2022) 11. doi: 10.7554/elife.74489 35587364 PMC9239683

[8] Sawant JJ . Structural and functional dissection of neutralisation differences among SARS-CoV-2 variants using antigenicity prediction and CR3022 binding analysis. (2026) 17:. doi: 10.3389/fimmu.2026.1810369 PMC13381479 42519305

[9] Dan JM . Immunological memory to SARS-CoV-2 assessed for up to 8 months after infection. Science. (2021) 371. doi: 10.1126/science.abf4063 33408181 PMC7919858

[10] Wehbe R . Emerging SARS-CoV-2 variants: Genomic shifts, immune evasion, and therapeutic perspectives. Mol Biol Rep. (2025) 52:886. doi: 10.1007/s11033-025-11009-w 40928692

[11] Uraki R . SARS-CoV-2 variants: Biology, pathogenicity, immunity and control. Nat Rev Microbiol. (2026) 24:8–28. doi: 10.1038/s41579-025-01255-x 41214236 PMC12973737

[12] Zhang A . Beyond neutralization: Fc-dependent antibody effector functions in SARS-CoV-2 infection. Nat Rev Immunol. (2023) 23:381–96. doi: 10.1038/s41577-022-00813-1 36536068 PMC9761659

[13] Clark JJ . Protective effect and molecular mechanisms of human non-neutralizing cross-reactive spike antibodies elicited by SARS-CoV-2 mRNA vaccination. Cell Rep. (2024) 43:114922. doi: 10.1016/j.celrep.2024.114922 39504245 PMC11804229

[14] Izadi A Nordenfelt P . Protective non-neutralizing SARS-CoV-2 monoclonal antibodies. Trends Immunol. (2024) 45:609–24. doi: 10.1016/j.it.2024.06.003 39034185

[15] Zhang J . Fc effector functions in RNA viral infections: Mechanisms of antiviral immunity and implications for vaccine design. Front Immunol. (2026) 17:1772257. doi: 10.3389/fimmu.2026.1772257 42199422 PMC13199346

[16] Chen X . The development and kinetics of functional antibody-dependent cell-mediated cytotoxicity (ADCC) to SARS-CoV-2 spike protein. Virology. (2021) 559:1–9. doi: 10.1016/j.virol.2021.03.009 33774551 PMC7975276

[17] Chen X . Fc-effector functional antibody assays for SARS-CoV-2 variants of concern. Front Immunol. (2025) 16:1571835. doi: 10.3389/fimmu.2025.1571835 40463385 PMC12130042

[18] Yu WH . ADCC-mediated CD56(DIM) NK cell responses are associated with early HBsAg clearance in acute HBV infection. Pathog Immun. (2018) 3:2–18. doi: 10.20411/pai.v3i1.228 29541698 PMC5847299

[19] Tomescu C Tebas P Montaner LJ . IFN-alpha augments natural killer-mediated antibody-dependent cellular cytotoxicity of HIV-1-infected autologous CD4+ T cells regardless of major histocompatibility complex class 1 downregulation. AIDS. (2017) 31:613–22. doi: 10.1097/qad.0000000000001380 28225449 PMC5934752

[20] Von Holle TA Moody MA . Influenza and antibody-dependent cellular cytotoxicity. Front Immunol. (2019) 10:1457. doi: 10.3389/fimmu.2019.01457 31316510 PMC6611398

[21] Hagemann K . Natural killer cell-mediated ADCC in SARS-CoV-2-infected individuals and vaccine recipients. Eur J Immunol. (2022) 52:1297–307. doi: 10.1002/eji.202149470 35416291 PMC9087393

[22] Yu Y . Antibody-dependent cellular cytotoxicity response to SARS-CoV-2 in COVID-19 patients. Signal Transduct Target Ther. (2021) 6:346. doi: 10.1038/s41392-021-00759-1 34561414 PMC8463587

[23] Gomez-Roman VR . A simplified method for the rapid fluorometric assessment of antibody-dependent cell-mediated cytotoxicity. J Immunol Methods. (2006) 308:53–67. 10.1016/j.jim.2005.09.018 16343526

[24] Dhande JR Bagul RD Thakar MR . HIV-gp140-specific antibodies generated from Indian long-term non-progressors mediate potent ADCC activity and effectively lyse reactivated HIV reservoir. Front Immunol. (2022) 13:844610. doi: 10.3389/fimmu.2022.844610 35309295 PMC8924355

[25] Motsoeneng BM . Despite delayed kinetics, people living with HIV achieve equivalent antibody function after SARS-CoV-2 infection or vaccination. Front Immunol. (2023) 14:1231276. doi: 10.3389/fimmu.2023.1231276 37600825 PMC10435738

[26] Alrubayyi A . Characterization of humoral and SARS-CoV-2 specific T cell responses in people living with HIV. Nat Commun. (2021) 12:5839. doi: 10.1038/s41467-021-26137-7 34611163 PMC8492866

[27] Alcaide ML . A longitudinal analysis of SARS-CoV-2 antibody responses among people with HIV. Front Med (Lausanne). (2022) 9:768138. doi: 10.3389/fmed.2022.768138 35330585 PMC8940197

[28] Feng Y . Immunogenicity of an inactivated SARS-CoV-2 vaccine in people living with HIV-1: A non-randomized cohort study. EClinicalMedicine. (2022) 43:101226. doi: 10.1016/j.eclinm.2021.101226 34901799 PMC8642727

[29] Ackerman ME . Natural variation in Fc glycosylation of HIV-specific antibodies impacts antiviral activity. J Clin Invest. (2013) 123:2183–92. doi: 10.1172/jci65708 23563315 PMC3637034

[30] Scully E Alter G . NK cells in HIV disease. Curr HIV/AIDS Rep. (2016) 13:85–94. doi: 10.1007/s11904-016-0310-3 27002078 PMC4821863

[31] Bick MV . Molecular parameters governing antibody FcgammaR signaling and effector functions in the context of HIV envelope. Cell Rep. (2025) 44:115331. doi: 10.1016/j.celrep.2025.115331 40158219 PMC12815480

[32] Krause R . HIV skews the SARS-CoV-2 B cell response towards an extrafollicular maturation pathway. Elife. (2022) 11. doi: 10.7554/elife.79924 36300787 PMC9643005

[33] Spinelli MA . SARS-CoV-2 seroprevalence, and IgG concentration and pseudovirus neutralising antibody titres after infection, compared by HIV status: A matched case-control observational study. Lancet HIV. (2021) 8:e334–41. doi: 10.1016/s2352-3018(21)00072-2 33933189 PMC8084354

[34] Karim F . HIV status alters disease severity and immune cell responses in Beta variant SARS-CoV-2 infection wave. Elife. (2021) 10. doi: 10.7554/elife.67397 34608862 PMC8676326

[35] Sondergaard MH . SARS-CoV-2 vaccine immunogenicity for people living with HIV: A systematic review and meta-analysis. HIV Med. (2024) 25:16–37. 10.1111/hiv.13537 37731375

